# Mechanisms of Hepatitis B Virus cccDNA and Minichromosome Formation and HBV Gene Transcription

**DOI:** 10.3390/v16040609

**Published:** 2024-04-15

**Authors:** Andoni Gómez-Moreno, Alexander Ploss

**Affiliations:** Department of Molecular Biology, Princeton University, Princeton, NJ 08544, USA

**Keywords:** hepatitis B virus, HBV, viral hepatitis, cccDNA, DNA repair, viral transcription

## Abstract

Hepatitis B virus (HBV) is the etiologic agent of chronic hepatitis B, which puts at least 300 million patients at risk of developing fibrosis, cirrhosis, and hepatocellular carcinoma. HBV is a partially double-stranded DNA virus of the *Hepadnaviridae* family. While HBV was discovered more than 50 years ago, many aspects of its replicative cycle remain incompletely understood. Central to HBV persistence is the formation of covalently closed circular DNA (cccDNA) from the incoming relaxed circular DNA (rcDNA) genome. cccDNA persists as a chromatinized minichromosome and is the major template for HBV gene transcription. Here, we review how cccDNA and the viral minichromosome are formed and how viral gene transcription is regulated and highlight open questions in this area of research.

## 1. Introduction

HBV is one of the most common human pathogenic viruses causing both acute and chronic hepatitis [1]. An estimated 2 billion people have been exposed to HBV, of whom 250–400 million are chronically infected, and ~800,000 individuals die each year from HBV-related liver diseases including decompensated cirrhosis and hepatocellular carcinoma [2,3]. HBV is an enveloped, non-cytolytic virus belonging to the *Hepadnaviridae* family [4]. HBV exclusively replicates in quiescent hepatocytes [5] by reverse transcription of an RNA intermediate, known as pregenomic RNA (pgRNA) [6]. First, virions attach to heparan sulfate proteoglycans (HSPGs) present on the surface of the hepatocytes [7]. Thereafter, the preS1 domain of the large envelope protein engages its host receptor, sodium taurocholate cotransporting polypeptide (NTCP) [8]. This highly specific interaction between preS1 and NTCP and the subsequent engagement of epidermal growth factor receptor (EGFR) [9] triggers HBV uptake via clathrin-mediated endocytosis [10]. Acidification of the endosome results in a conformational change in the viral envelope proteins, forcing fusion of the viral with the endosomal membrane, ultimately releasing the HBV genome-containing nucleocapsid into the cytoplasm. The capsid is subsequently transported to the nuclear pore complex using the microtubule network [11], and the HBV relaxed circular DNA (rcDNA) genome is released into the inner part of the nucleus. The exact mechanisms responsible for capsid disassembly and rcDNA release remain incompletely understood [12]. Here, rcDNA is repaired by host nuclear proteins into cccDNA, which is assembled into a chromatinized episome termed minichromosome [13] and serves as the major transcriptional template to produce viral RNAs. Viral mRNAs are produced by host RNA polymerase II (RNAPII) and, therefore, contain a 5′-capped structure and share a common 3′-end polyadenylation site. The specific interaction of viral polymerase with the 5′ ε stem-loop structure of pgRNA in the cytoplasm triggers the packaging of the pgRNA inside viral capsids where reverse transcription occurs [14]. In brief, the viral polymerase synthetizes the minus DNA strand via its RNA-dependent DNA polymerization activity using the pgRNA as the template. Simultaneously, the RNase H activity of the polymerase degrades the pgRNA. Finally, the DNA-dependent DNA polymerization activity of the polymerase generates the plus DNA strand using the minus strand as a template [15]. The reverse transcription process creates rcDNA molecules and double-stranded linear DNA (dslDNA) forms as a by-product that constitutes up to 10% of all viral DNA molecules [16]. The capsids harboring these newly reverse-transcribed genomes can be transported back to the nucleus, and contribute to the maintenance of the cccDNA pool, in what is known as the intracellular recycling pathway [17]. On the other hand, nascent nucleocapsids can associate with HBV surface proteins and acquire the envelope to produce infectious viral particles that are released from the cell via the secretory pathway and the formation of multivesicular bodies [18]. Along with infectious viral—also known as Dane—particles, infected cells also release soluble HBeAg and non-infectious subviral particles including hepatitis B surface antigen (HBsAg) (spheres and filaments) and naked empty capsids [19]. HBeAg and HBsAg release may be an immune evasion strategy during HBV infection [20,21]. It is important to highlight that HBV DNA can integrate into the host genome even though it is not required for the completion of its replicative cycle [22]. In this review, we will focus on cccDNA formation, minichromosome formation, transcription, and post-transcriptional modifications.

## 2. HBV cccDNA Formation

**Figure 1:**
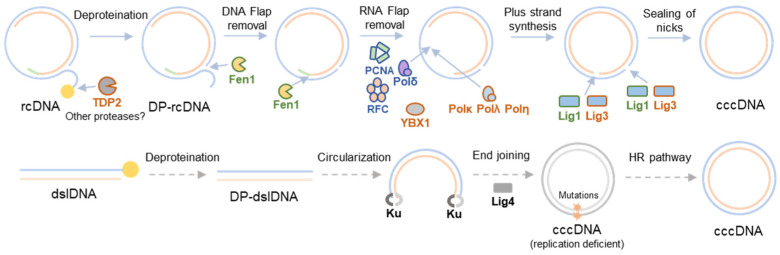
**Cellular host factors involved in cccDNA formation.** Host factors that have been identified *in vitro*, *in vivo*, or both are highlighted in blue, brown, and green, respectively. Two cccDNA repair pathways have been proposed, rcDNA to cccDNA (top panel) and dslDNA to cccDNA (bottom panel). RcDNA to cccDNA formation involves deproteination, DNA, and RNA flap removal, plus-strand synthesis, and sealing of both nicks. TDP2 and Fen1 are involved in the removal of the viral polymerase, and the DNA and RNA flap, respectively. PCNA and RFC together with POLκ, POLδ, POLη, and POLλ are involved in plus-strand synthesis. Host factor YBX1 may play a role during this step. Finally, plus- and minus-strand nicks are sealed by Lig1 and Lig 3. Of note, all these processes may occur in a different order or even simultaneously, and additional cellular proteins may play redundant roles *in vivo*. On the other hand, dslDNA to cccDNA formation involves deproteination, circularization, and homologous recombination (HR) steps. Circularization may be driven by cellular proteins involved in the NHEJ pathway such as Ku heterodimer (Ku) and ligase 4 (Lig 4). This repair process generates replication-deficient cccDNA molecules that may become replication-competent in a process that involves homologous recombination. It is important to highlight that this second pathway is based on the circularization of DHBV dslDNA by NHEJ, and as far as we know, it has not been demonstrated for HBV. Thus, all these steps are represented with dashed grey arrows.

RcDNA is a double-stranded, 3.2 kb long, circular DNA with distinct structural features reminiscent of its complex DNA replication cycle [4]. This molecule contains four different lesions including the covalently linked HBV polymerase (pol) linked via a 10 nucleotide (nt) DNA flap to the 5′-end of the minus strand, a 5′-capped RNA primer, and a single-stranded DNA (ssDNA) gap on the plus strand [23]. Therefore, conversion of rcDNA to cccDNA encompasses a set of processes that include the release of viral polymerase and removal of the redundancy terminal region from the minus strand, degradation of the RNA oligomer from the plus strand, completion of the plus strand, and ligation of both strands. Since the enzymes capable of catalyzing these relevant reactions needed to produce cccDNA from rcDNA are not encoded in the viral genome, rcDNA to cccDNA formation depends on host cellular proteins, as shown in Figure 1.

The first step involves the release of the viral pol from the rcDNA to produce deproteinated rcDNA (DP-rcDNA). Different cellular proteins such as tyrosyl-DNA phosphodiesterase 2 (TDP2), flap endonuclease 1 (FEN-1), or cellular proteases, may be involved in this process [24,25]. Biochemical studies revealed that core components of DNA lagging strand synthesis machinery, including proliferating cell nuclear antigen (PCNA), replication factor C (RFC), and polymerase δ (pol δ), Fen1, and DNA ligase 1 (Lig1), are sufficient to repair rcDNA into cccDNA in vitro [26]. Each strand is repaired via the following distinct mechanisms: negative strand repair only requires Fen1 and Lig1, while positive strand repair requires all five factors [27]. *In cellulo*, additional factors may be involved in this process. Results stemming from loss of function experiments in HBV-infected HepG2-hNTCP cells suggest a role for cellular DNA polymerase κ (POLκ) and, to a lesser extent, DNA polymerases POLη and POLλ in plus-strand completion [28]. Therefore, it is possible that cellular DNA polymerases serve redundant roles in cccDNA formation. Conceivably, additional cellular factors may influence HBV cccDNA formation efficiency, as evidenced by the identification of Y box binding protein 1 (YBX1), a DNA binding protein regulating transcription and translation, in a limited loss-of-function screen [29].

An alternative pathway for cccDNA formation has been proposed for duck hepatitis B virus (DHBV), and it is based on the circularization of double-stranded linear DNA (dslDNA) into cccDNA [30,31]. DslDNA is formed as a consequence of a priming failure during reverse transcription that leads to the synthesis of dslDNA instead of rcDNA [16]. Experiments carried out with DHBV in a CHO-derived cell line showed that Ku80, a sensory component of the non-homologous end joining (NHEJ) repair pathway, was necessary for cccDNA formation from dslDNA but not from rcDNA, suggesting that this pathway is involved in dlsDNA-derived cccDNA formation [32]. Deletions and insertions around dslDNA junctions have been detected in DHBV cccDNA molecules [30,31], which are also commonly found in chromosomal DNA repaired by the NHEJ pathway [33]. While these deletions and insertions do not enhance rcDNA biosynthesis, dslDNA-derived cccDNA can revert into wild-type cccDNA, possibly via homologous recombination [31]. This process is known in the field as illegitimate replication, a term coined by Jesse Summers [30]. Whether these mechanisms can result in the formation of functional cccDNA for human HBV is unknown. Comparatively, in human HBV, dslDNA is the predominant precursor for integration in the host genome [34]. For further reading about HBV dslDNA integration, we refer the reader to [22].

## 3. HBV Minichromosome Formation

HBV cccDNA persists in the nuclei of infected hepatocytes as a minichromosome associated with viral (e.g., HBX and HBV core) and cellular (e.g., histones and transcription factors) proteins [35]. Our knowledge of how different chromatin remodelers affect cccDNA minichromosome composition and impact cccDNA minichromosome expression, and how this influences HBV pathology, has substantially increased over the last years (reviewed in [36,37]). However, less is known about how these processes take place very early during infection. First, it is important to highlight that the rcDNA that is released into the inner part of the nuclei of infected hepatocytes is not associated with histone or nuclear transcription factors as it is produced in the cytoplasm by the reverse transcription of pgRNA [6]. Therefore, association with histones or transcription factors to the HBV genome necessarily begins in the nucleus. Whether this association is coupled with the rcDNA repair process, is a post-repair process, or both is unknown. Identification of the histone regulator A complex (HIRA) as a factor that supports HBV minichromosome establishment and transcriptional activity in infected hepatocytes suggests that histone deposition may be coupled to the rcDNA repair process [38]. HIRA promotes H3.3 deposition and nucleosome assembly by exploiting its capacity to bind nonspecifically to H3.3–H4 histone dimers on naked DNA [39,40], including foreign viral DNA [41]. This nucleosome assembly is promoted upon DNA damage in host chromatin [42]. Transfection of nicked minicircle HBV DNA and Southern blot assays showed that a nicked, partially double-stranded precursor is the substrate of HIRA during cccDNA formation [38]. However, these results do not discount the possibility that HIRA may play a role in rcDNA to cccDNA repair as part of its role in HBV minichromosome assembly. Moreover, the transfection of plasmids encoding full-length HBV genome and H3.3 ChIP assays performed in HIRA-silenced cells suggested that HIRA histone deposition also occurred in fully repaired DNA templates [43]. Further experiments will be required to precisely decipher the molecular mechanisms of the association between host nuclear proteins and HBV cccDNA.

Other players contributing to HBV minichromosome formation at the early stages of infection are HBV viral proteins. Particularly, HBV proteins that constitute the nucleocapsid, i.e., viral polymerase and core. These viral proteins are transported along with rcDNA into the inner part of the nuclei. Both HBV core and pol are not necessary for cccDNA formation since transfection of recombinantly produced RrcDNA into HepG2 cells supports cccDNA conversion in the absence of core protein [26], and highly potent and selective HBV pol inhibitors revealed that HBV pol activity is not necessary for cccDNA formation [28]. Less is known about whether these proteins may play a role in the early stages of minichromosome formation, including rcDNA to cccDNA repair. Particularly, virion-delivered HBV core protein can associate with cccDNA in the absence of viral transcription and de novo protein synthesis, and this viral association is stable for at least two weeks after infection [44]. In this context, HBV core interacts with the HIRA complex and other chromatin remodelers [38,45]; therefore, it is not surprising that virion-delivered core may play a role in minichromosome formation and even cccDNA formation. Further analysis is needed to decipher the role (if any) of virion-delivered core on minichromosome formation.

## 4. Viral Gene Expression from the HBV Minichromosome

Once the HBV minichromosome is formed, cellular RNAPII is responsible for the production of viral mRNAs. All viral transcripts contain a 5′-capped structure and share a common 3′-end polyadenylation site. Transcription is driven by four viral promoters (preS1, preS2, core, and X) and two enhancers (Enh1 and Enh2) that are distributed throughout the viral genome and produce six canonical unspliced transcripts that encode a total of seven viral proteins as follows: the viral polymerase (pol) and core (HBcAg) proteins are produced from the pgRNA; the pre-core protein (e antigen or HBeAg) from the pre-core mRNA; the large (L), medium (M), and small (S) envelope surface proteins from the PreS1, PreS2, and M/S mRNAs, respectively; and HBx protein from the X mRNA. Because of the circular nature of HBV cccDNA, more than one-unit length sequence mRNAs are generated (e.g., pre-core and pgRNA), and there is overlap in all viral mRNAs. This overlapping nature of HBV mRNAs, together with the high diversity of other non-canonical mRNAs produced during HBV infection, has been a bottleneck to studying the expression profiles of different HBV mRNA expressed during infection. In this context, long-read RNA sequencing has emerged as a promising tool for the characterization of the HBV transcriptome [46,47].

This considerable heterogeneity may arise from chimeric (viral and cellular) mRNAs produced as a consequence of the integration of partial HBV genome sequences into the host chromosomal DNA and specifically because of the different splicing processes on HBV mRNA. More than 20 different splice variants have been described so far as a consequence of alternative splicing processes [48,49]. Three splice variants can yield non-canonical viral proteins whose role is currently being studied [50,51,52].

On the other hand, as viral transcription depends on the cellular machinery, it is regulated by host epigenetic mechanisms. Therefore, several histone modifiers including histone acetyltransferase 1 (HAT-1), histone-lysine N-methyltransferase SETDB1, arginine methyltransferase (PRMT1), and cellular structural maintenance of chromosome complex 5/6 (Smc5/6), as well as host DNA methylases and other chromatin remodelers mentioned before, can regulate HBV cccDNA transcription [38,45,53,54,55,56,57] (reviewed in [37]).

Finally, we would like to highlight that the spatial localization of HBV cccDNA in the nucleus also contributes to regulating cccDNA transcription. The three-dimensional (3D) architecture of chromosomes and their localization within nuclei is known to play an important role in cellular transcription processes [58]. Proximity ligation 4C-seq and fluorescent *in situ* hybridization (FISH) analysis targeting HBV cccDNA suggest that cccDNA is not randomly distributed in the nucleus. Transcriptionally active cccDNA localizes in transcriptionally active chromatin regions, while transcriptionally inactive cccDNA is preferentially localized in chromosome 19 [59]. Changes in nuclear HBV cccDNA distribution are primarily controlled by HBx protein expression and the host Smc5/6 restriction complex. In fact, it is known that the Smc5/6 complex restricts HBV expression when localized to nuclear domain 10 (ND10) in primary human hepatocytes [60], and a recent study demonstrated that transcriptionally inactive HBx-deficient cccDNA is recruited to promyelocytic leukemia nuclear bodies (PML-NBs) via direct interactions with the Smc5/6 complex mediated by Smc5–Smc6 localization factor 2 (SLF2) [61,62]. On the other hand, it has been recently shown that G-quadruplexes, secondary nucleic acid structures [63], are present in HBV cccDNA and mediate phase separation promoting HBV cccDNA transcription [64]. Given that the Smc5/6 complex shows a preference for binding supercoiled and catenated DNA templates [65], it would be intriguing to investigate whether the presence of various G-quadruplexes in HBV cccDNA affects its recognition by the Smc5/6 complex.

## 5. Role of HBV Proteins in cccDNA Transcription

Once the HBV minichromosome is established and transcriptionally active, the translated viral proteins can be transported to the nucleus and modulate cccDNA minichromosome composition and, therefore, its expression. The following two viral proteins have been shown to be associated with the HBV minichromosome: HBx and core [35,66]. We already discussed the possible role of virion-delivered core on cccDNA minichromosome formation. Nevertheless, it is important to highlight that a new synthesis of core is not required for cccDNA transcriptional regulation. This is supported by several lines of evidence provided by two independent laboratories. The infection of cells with wild-type (wt) and core-deficient HBV mutants [67], as well as transfection of *in vitro*-produced wild-type and core-deficient HBV circle DNA [68], was shown to result in equivalent amounts of cccDNA, mRNA, and intracellular and secreted HBsAg. It should be noted that these studies were conducted over a fairly short period of 1–2 weeks, but the results argue against a possible role of the newly synthesized core in cccDNA expression and further suggest a minor contribution of the intracellular recycling of newly formed rcDNA containing capsids to the maintenance/increase in the existing nuclear cccDNA pool. Thus, initially formed cccDNA may indeed be stable in hepatocytic cells without necessitating continuous replenishment in in vitro infection systems. On the other hand, there is robust evidence from clinical studies and from human liver chimeric mice that the process is indeed more dynamic and that the cccDNA pool is constantly replenished [69,70].

**Figure 2:**
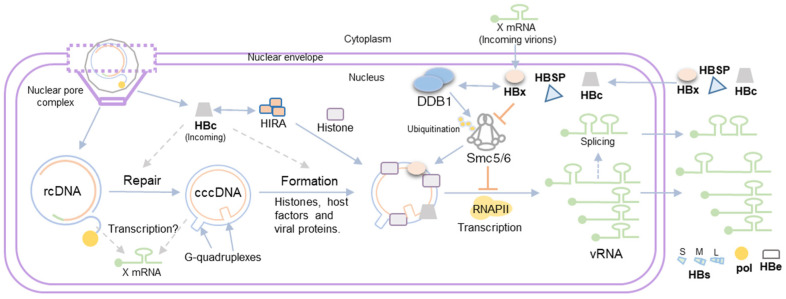
**Role of viral proteins in minichromosome transcription.** Steps in the viral replicative cycle including rcDNA release, repair into cccDNA, minichromosome assembly, transcription, and translation are represented. (Incoming) HBc released from the viral nucleocapsid may recruit chromatin remodelers such as the HIRA complex and, therefore, might have an impact on minichromosome formation as well as rcDNA to cccDNA repair in the early steps post-infection. On the other hand, while newly synthesized HBc can be transported to and accumulate in the nucleus, it is not required for cccDNA transcriptional regulation. The HBx protein mediates the degradation of the Smc5/6 complex by interaction with DDB1. Two hypotheses of how the HBx protein, which is not included in the viral particle, arises before Smc5/6 complex restriction are represented. First, HBx RNA may be packaged in viral particles and can be readily translated upon infection. Second, rcDNA lesions or G-quadruplexes present in the cccDNA may not be recognized by the Smc5/6 complex, allowing for HBx RNA transcription at very early stages. Thus, the HBx protein is translated from this first round of transcription and the Smc5/6 complex is degraded, allowing for a second round of transcription. Other HBV proteins, generated from spliced RNAs such as HBV spliced protein (HBSP), are nuclear, and their role is now being studied.

HBx is a nuclear protein that is expressed very early and at low levels during infection [71]. Its role is to promote the degradation of the Smc5/6 complex, a restriction factor of any episomal DNA expression [72], including HBV cccDNA [56]. For this reason, the HBx protein plays a central role in cccDNA expression. HBx transactivation activity relies on its ability to bind to DNA damage binding protein 1 (DDB1) [73]. DDB1 interacts with cullin 4 (cul4) as part of an E3 ubiquitin ligase complex responsible for the polyubiquitination and proteasome-dependent degradation of target proteins [74]. Thus, HBx hijacks the DDB1-cul4 protein complex system to mediate the degradation of Smc5/6, thereby allowing viral transcription from cccDNA. Because the HBx protein is not known to be packaged in HBV infectious particles, it is puzzling how this viral protein manages to facilitate Smc5/6 complex degradation to counter the repression of viral gene transcription by this complex. Several potential explanations have been proposed as follows: Full-length 5′ rapid amplification of cDNA ends (RACE) analysis revealed that in HBV viral particles containing pgRNA, pgRNA splice variants, and different HBx RNAs are present in HBV-infected hepatocytes and patient serum [75]. Thus, encapsidated HBx RNA present within HBV infectious particles may be readily translated without the need for *de novo* HBx RNA transcription off newly formed cccDNA. Another possibility is that HBx transcripts may arise from non-repaired rcDNA or non-chromatinized cccDNA templates that may not be restricted by the Smc5/6 complex. In this context, it is still not clear how the Smc5/6 complex recognizes and binds to episomal DNA. As aforementioned, the Smc5/6 complex preferentially binds supercoiled over relaxed DNA and may compact supercoiled DNA (i.e., transcriptionally active DNA) [65,76]. Therefore, it is conceivable that at very early stages during rcDNA release into the nucleus, the Smc5/6 complex may not be able to recognize viral DNA, thus allowing HBx mRNA transcription and, consequently, Smc5/6 complex degradation prior to Smc5/6 complex transcriptional repression. The role of HBx as well as other viral and cellular proteins in minichromosome transcription is summarized in Figure 2.

## 6. HBV Post-Transcriptional Modifications

Over the past few years, it has become clear that RNA is heavily modified post-transcriptionally and that these epitranscriptomic modifications have profound effects on RNA homeostasis [77]. Eukaryotic cellular RNAs contain diverse chemical modifications [78], with methylation of the adenosine base at the nitrogen 6 position (m^6^A) being the most common and the best-characterized modification of cellular RNAs [79]. HBV RNAs are produced by host RNAPII in the nucleus, exported to the cytoplasm, and, consequently, are subject to different post-transcriptional modifications in both compartments.

As mentioned in the preceding sections, synthesis of HBV transcripts is initiated from different transcription start sites in the HBV genome, but it terminates at a common 3′ transcription end site. All the HBV viral transcripts have a common 3′ epsilon stem-loop, and pgRNA and pre-core mRNA have the same epsilon stem-loop duplicated in the 5′. This epsilon stem-loop is subject to m^6^A methylation, and this RNA modification differentially regulates the viral life cycle depending on its position in the viral RNAs. m^6^A modification at the 3′ epsilon stem-loop of HBV RNA transcripts reduces their RNA stability, leading to decreased viral protein expression [80]. On the other hand, the m^6^A site located in the 5′ epsilon stem-loop of pgRNA is necessary for pgRNA encapsidation as it promotes the interaction between pgRNA and core protein [81]. Conceivably, splice transcripts harboring the 5′ epsilon stem-loop may also be encapsidated and, consequently, secreted. Epsilon stem loops also play an important role during reverse transcription of encapsidated pgRNA but interestingly, interactions between HBV polymerase and 5′ epsilon are independent of the m^6^A modification of 5′ epsilon [81].

Moreover, previous studies have demonstrated that HBx interacts with methyltransferase complex (METTL) 3/14, thereby transporting these m^6^A methyltransferases to HBV cccDNA for co-transcriptional methylation of HBV RNAs [82]. These results underscore a new function of HBx during HBV infection, aiding in cotranscriptional RNA modification alongside its involvement in cccDNA transactivation and its impact on the Smc5/6 complex and HBx-DDB1-mediated degradation activity.

Finally, HBV pgRNA deamination by enzymes belonging to the apolipoprotein B editing complex (APOBEC) family, such as APOBEC3G or activated induced cytosine deaminase (AID), has been suggested [83,84]. However, other studies failed to detect pgRNA deamination, suggesting that deamination occurs after reverse transcription of pgRNA [85].

## 7. Concluding Remarks

It is widely accepted that any attempt to cure chronic hepatitis B will necessitate permanently inactivating or eliminating cccDNA from infected cells. To achieve this goal a detailed understanding of how cccDNA and, subsequently, the minichromosome are formed and HBV genes are transcribed is critical. Much has been learned over the past decades about these intricate processes from work by numerous laboratories around the globe, but some knowledge gaps remain.

The formation of HBV cccDNA involves intricate processes that rely heavily on host cellular proteins. The conversion of rcDNA to cccDNA necessitates a series of steps, catalyzed by numerous host factors that are not yet fully defined, some of which may serve redundant functions. Comparing how cccDNA is formed across different members of the *Hepadnaviridae* family may prove to provide additional insights.

The transcription of HBV genes from the cccDNA minichromosome is regulated by host epigenetic mechanisms, with histone modifiers and chromatin remodelers playing crucial roles. The development of biochemical tools will be helpful in further dissecting the relative contributions of these factors systematically. It remains unknown how genetic variation in different HBV genotypes and intra-genotypic polymorphisms affect HBV gene transcription. Moreover, the spatial localization of cccDNA within the nucleus significantly impacts its transcriptional activity, as actively transcribed cccDNA tends to associate with transcriptionally active chromatin regions. Investigating whether and how HBV DNA integrated into the host genome potentially influences this regulation will be a crucial future direction. Advanced chromosome conformation capture techniques, allowing unbiased genome-wide mapping of all occurring interactions, will undoubtedly contribute to gaining further insights into this regulatory mechanism.

Post-transcriptional modifications of HBV RNAs, particularly m^6^A methylation, modulate viral RNA stability and protein expression. However, hundreds of other epitranscriptomic modifications have been described, of which a dozen or so have been detected in cells. As existing detection tools are being refined, and new ones are developed a more complete picture of other modifications on HBV RNAs will emerge, and their functions can be subsequently elucidated.

Such insights may provide valuable targets for the development of novel therapeutic strategies against chronic HBV infection.

## Data Availability

Not applicable.
